# Creating animal models, why not use the Chinese tree shrew (*Tupaia belangeri chinensis*)?

**DOI:** 10.24272/j.issn.2095-8137.2017.032

**Published:** 2017-05-18

**Authors:** Yong-Gang Yao

**Affiliations:** ^1^Key Laboratory of Animal Models and Human Disease Mechanisms, Kunming Institute of Zoology, Chinese Academy of Sciences, Kunming Yunnan 650223, China; ^2^Kunming Primate Research Center of the Chinese Academy of Sciences, Kunming Institute of Zoology, Chinese Academy of Sciences, Kunming Yunnan 650223, China

**Keywords:** Chinese tree shrew, Genome biology, Animal model, Gene editing, Innate immunity

## Abstract

The Chinese tree shrew (*Tupaia belangeri chinensis*), a squirrel-like and rat-sized mammal, has a wide distribution in Southeast Asia, South and Southwest China and has many unique characteristics that make it suitable for use as an experimental animal. There have been many studies using the tree shrew (*Tupaia belangeri*) aimed at increasing our understanding of fundamental biological mechanisms and for the modeling of human diseases and therapeutic responses. The recent release of a publicly available annotated genome sequence of the Chinese tree shrew and its genome database (www.treeshrewdb.org) has offered a solid base from which it is possible to elucidate the basic biological properties and create animal models using this species. The extensive characterization of key factors and signaling pathways in the immune and nervous systems has shown that tree shrews possess both conserved and unique features relative to primates. Hitherto, the tree shrew has been successfully used to create animal models for myopia, depression, breast cancer, alcohol-induced or non-alcoholic fatty liver diseases, herpes simplex virus type 1 (HSV-1) and hepatitis C virus (HCV) infections, to name a few. The recent successful genetic manipulation of the tree shrew has opened a new avenue for the wider usage of this animal in biomedical research. In this opinion paper, I attempt to summarize the recent research advances that have used the Chinese tree shrew, with a focus on the new knowledge obtained by using the biological properties identified using the tree shrew genome, a proposal for the genome-based approach for creating animal models, and the genetic manipulation of the tree shrew. With more studies using this species and the application of cutting-edge gene editing techniques, the tree shrew will continue to be under the spot light as a viable animal model for investigating the basis of many different human diseases.

## INTRODUCTION

As human beings, our knowledge about ourselves, especially about how our brain works, how a disease develops, and the discovery of many efficient therapeutic agents, has largely come from studies using animals. The higher the similarity between an animal species and the human, the more we can obtain helpful and precise information concerning the fundamental biology, disease mechanism, and safety, efficiency and predictability of therapeutic agents (Franco, 2013; McGonigle & Ruggeri, 2014). Because of ethical concerns and restrictions, chimpanzees and large primates have been forbidden from being used in the creation of most animal models and in many types of drug tests, albeit they remain the best animals for studying human physiology (Bennett & Panicker, 2016; Knight, 2008). Monkeys have played a critical role in medical research (Zhang et al., 2014), but their costs are relatively high. Rodents are more commonly used in biomedical research, however, the results are sometimes difficult to extrapolate due to species disparity, methodological flaws and other reasons (van der Worp et al., 2010). The search for a suitable animal that is close to the human, but with a modest cost-efficiency, for use as a model for the study of disease is a long pursued task. On the other hand, each species has its own unique features, and we need to understand more about the particular species before we can attempt to use it in biomedical research.

The tree shrew (*Tupaia belangeri*) is a squirrel-like and ratsized mammal that is widely distributed in Southeast Asia, South and Southwest China. It has a small body size (100-150 g), a low-cost of maintenance, a short reproductive cycle (~ 6 weeks) and life span (6-8 years), a high brain-to-body mass ratio, and a close relationship to primates (Peng et al., 1991; Zheng et al., 2014). In the past few decades, the tree shrew has been used in biomedical research to increase our understanding of the fundamental biological and pathological mechanisms of life and disease (Amako et al., 2010; Cao et al., 2003; Fitzpatrick, 1996; Fuchs, 2005; Muly & Fitzpatrick, 1992; Peng et al., 1991; Su et al., 1987; Xu et al., 2013b; Yan et al., 1996; Zhao et al., 2002; Zheng et al., 2014). There is a proposal for using the tree shrew to replace primates in biomedical research (Cao et al., 2003; Peng et al., 1991), albeit there is still a long way to go.

## THE CLOSE GENETIC RELATIONSHIP OF THE TREE SHREW TO PRIMATES

The phylogenetic relationship of the tree shrew in the Euarchontoglires has been debated for a long time and a clarification of the genetic relationship of the tree shrew to primates will provide a firm basis for using the tree shrew as an alternative to primates in biomedical research. Previous studies have reported different clustering patterns regarding the phylogenetic affinity of the tree shrew to primates, lagomorphs and rodents on the basis of various kinds of genetic data (Fan et al., 2013; Lin et al., 2014; O'Leary et al., 2013; Song et al., 2012; Xu et al., 2012, 2013a; Zhou et al., 2015). The recent comparative genome analysis of the Chinese tree shrew (*Tupaia belangeri chinensis*) and related vertebrate species (including primates) has provided sufficient evidence to resolve this question and has showed that the tree shrew has a much closer affinity to primates than that of rodents (Fan et al., 2013; Lin et al., 2014; Xu et al., 2013a). Note that a recent phylogenomic analysis of 1 912 exons from 22 representative mammals claimed that the position of tree shrews within the Euarchonta is unstable (Zhou et al., 2015). Leaving aside the technical problems and the usage of different datasets, the current taxonomical status of tree shrew in the Order Scandentia is well supported (Fan et al., 2013; Xu et al., 2013a).

## COMMON AND UNIQUE GENETIC PROPERTIES OF THE TREE SHREW

Despite the closer affinity of the tree shrew to primates as compared to that of rodents to primates, the estimated divergence time between the tree shrew and primates has been estimated to be around 90.9 million years ago (Fan et al., 2013). This lengthy period of time has resulted in the evolution of a number of unique genetic features in the tree shrew, whilst many other genetic features have continued to be present in both the tree shrew and in primates.

### Common genetic properties between the tree shrew and human

Our previous comparative genome analysis of the tree shrew and human identified 28 genes previously considered to be primate-specific in the tree shrew genome, and there was a high sequence identity between the tree shrew and human for the majority of those genes/pathways involved in neuropsychiatric disorders and infectious diseases (Fan et al., 2013). For instance, we found all the human neurotransmitter transporters in the tree shrew genome, and these gene sequences were highly conserved between the tree shrew and human (Fan et al., 2013). Neuropeptidomics of the brain tissue of tree shrews showed that the identified neuropeptides have a significantly higher degree of homology to the equivalent sequences in humans than those in rodents (Petruzziello et al., 2012). This genetic pattern was compatible with the usage of the tree shrew as an experimental model for studying psychosocial stress and antidepressant drug effect (Fuchs, 2005; Pryce & Fuchs, 2017). The genes in the Aβ production and neurofibrillary tangles formation pathways, which produce the two hallmarks of Alzheimer's disease (AD), also had a generally higher sequence identity with human (unpublished data). In particular, the Aβ42 peptide sequence of tree shrew was the same as that of human, whereas mouse and rat all differed from human by three residues (Pawlik et al., 1999). The gene expression pattern of the AD-related genes in the Chinese tree shrew also resembled that of rhesus monkey (*Macaca mulatta*) and human, but differed remarkably from that of mouse (unpublished data). These observations have suggested that the tree shrew has the genetic basis for being used to create an AD model, and could also explain previous observations for an early stage of amyloid accumulation in the brains of aged tree shrews (Yamashita et al., 2010, 2012). The majority of the autism-related genes also shared a high level of sequence identity (with less than 5% of variance compared to human) between the tree shrew and human (Fan & Yao, 2014). An analysis for common candidate drug targets, such as kinases, G-protein-coupled receptors, ion channel proteins, nuclear receptors, immune-related proteins, neuropeptides, proteases and inhibitors, in the Chinese tree shrew showed that half of the predicted drug targets had a higher sequence similarity to human targets than found in the mouse (Zhao et al., 2014). Also, proteomic characterization of tree shrew liver and muscle tissues demonstrated that nearly half of the identified proteins were highly similar to those of humans, whereas only 25% of them were highly similar to rats or mice, suggesting that the tree shrew is closer to the human than to the mouse and rat (Li et al., 2012). All these pieces of evidence of a high level of similarity in gene sequences, pathway components and proteomic characteristics between the tree shrew and the human have laid the foundation for an essential genetic basis to use the tree shrew in the creation of a viable animal model to study these related diseases.

### Unique genetic differences between the tree shrew and human

The unique genetic features of the tree shrew provide a good opportunity for us to understand the specific physiobiology of the tree shrew and to explore the biological implications of adaptation and evolution. For instance, among the 209 known visually related human genes, the tree shrew only lacked the *OPN1MW* (opsin 1 (cone pigments), medium-wave-sensitive) and *OPN1NW2* (opsin 1 (cone pigments), medium-wavesensitive 2) genes. The lack of these two cone photoreceptor genes, as well as other unique features in the rod photoreceptor rhodopsin (Fan et al., 2013), are compatible with the diurnal pattern of behavior and dichromacy of the tree shrew (Hunt et al., 1998). The evolutionary comparison of 407 locomotion system related genes in human, monkey, tree shrew, dog, rat and mouse showed that 29 genes had undergone positive selection, including *HOXA6* (homeobox A6) and *AVP* (arginine vasopressin) that affected skeletal morphogenesis or muscle contraction (Fan et al., 2014b). This observation is compatible with the tree shrew's ability to move fast and jump strongly (Fan et al., 2014b).

**Figure 1:**
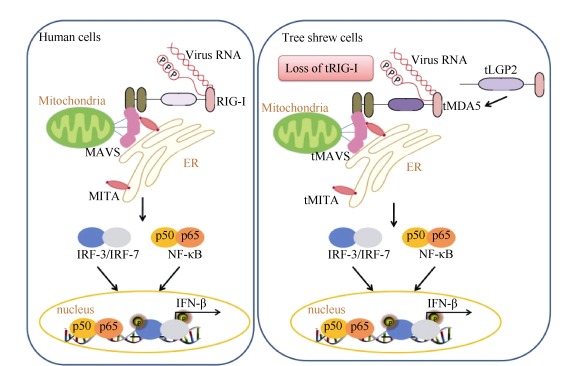
Schematic profile of the antiviral response in the Chinese tree shrew (right) and human (left)

The absence of certain genes (relative to human) from the tree shrew genome provides a model to understand specific pathways that were mediated by these lost genes. In the previous analysis, we have provided a list of 11 examples of (potential) gene loss and 144 pseudo-genes in the tree shrew genome (Fan et al., 2013); and these are now undergoing further validation and characterization studies. Among these genes, loss of the important antiviral gene *RIG-I* (retinoic acidinducible gene Ⅰ, also known as DDX58) in the Chinese tree shrew lineage has provided us a rare opportunity to understand the evolutionary adaptation and functional diversity of antiviral activity in vertebrates. Moreover, this might be one of the principal genetic reasons for the tree shrew's suitability as an animal model for studying viral infections. Previous intensive studies of innate immunity showed that after viral challenge, the pattern recognition receptors (PRRs) had a rapid response, leading to the subsequent production of antiviral cytokines such as type-I interferons (IFNs), inflammatory factors and complements (Takeuchi & Akira, 2010). These cytosolic PRRs, including Tolllike receptors (TLRs), NOD-like receptors (NLRs), RIG-I-like receptors (RLRs) and cytosolic DNA receptors, are actively involved in the host innate immunity response against invasion by pathogens (Barbalat et al., 2011). The RLRs contains three members, RIG-I, MDA5 (melanoma differentiation factor 5, also known as IFIH1), and LGP2 (laboratory of genetics and physiology 2, also known as DHX58), which are found in the cytosol of most types of mammalian cells and act as PRRs to response to viral RNA (Barbalat et al., 2011; Takeuchi & Akira, 2010). The loss of RIG-I in the Chinese tree shrew has raised several interesting questions concerning the unique genetic features of the innate immune response in this animal and the evolution of innate immunity in mammals: why can the *RIG-I* gene be lost in this animal? Is there a functional replacement for this key factor in the tree shrew immune system? What are the biological implications concerning this evolutionary event of the antiviral innate immunity in mammals? An understanding of the biological effects of the loss of RIG-I in the Chinese tree shrew will undoubtedly offer insights into the origin and development of the innate immunity in mammals. Recently, we attempted to answer these questions and deciphered the mechanisms underlying the loss of *RIG-I* in the Chinese tree shrew (Xu et al., 2016). We first confirmed the absence of *RIG-I* in the tree shrew lineage by analyzing a group of tree shrews collected from different regions and by comparing them to the Malayan flying lemur (which had a close relationship to the tree shrew). Further viral infection tests showed that the loss of this gene did not impair *IFNB1* induction in the tree shrew primary renal cells in response to a variety of RNA viruses, indicating that there is a functional substitute or a compensation of the response network for the loss of RIG-I in sensing viral RNAs in the tree shrew. Alongside the loss of *RIG-I*, both *MDA5* (*tMDA5*) and *LGP2* (*tLGP2*) had undergone strong positive selection in the tree shrew. Moreover, tMDA5 or tMDA5/tLGP2 could sense Sendai virus (a RNA virus used as a RIG-I agonist) by inducing IFN, though conventional RIG-I and MDA5 were thought to recognize distinct RNA structures and viruses. tMDA5 also acquired an ability to interact with adaptor tMITA (STING/ TMEM173/ERIS), which was reported to bind only with RIG-I. Further functional analysis showed that the positively selected sites (PSSs) in tMDA5 endowed the substitute function for the lost RIG-I (Xu et al., 2016). The evolutionary interpretation of the potential function of the PSSs in the tree shrew was further supported by a gain-of-function analysis, in which we introduced the positively selected variants at the equivalent positions in human MDA5. These artificially made human MDA5 mutants showed an enhanced antiviral function (Xu et al., 2016). As a consequence of working on the evolutionary event of *RIG-I* loss in the tree shrew, we were able to uncover a previously unknown evolutionary signal in response to RIG-I loss in the tree shrew (Xu et al., 2016). This special case provided insights into the functional conservation and divergence of RLRs in innate immunity (Figure 1).

To give another example of the genetic uniqueness of the tree shrew immune system, we recently characterized the evolution of the tTLRs in the Chinese tree shrew. We found that the tree shrew had 13 putative TLRs (including 12 orthologs of mammalian *TLR1*-*TLR9* and *TLR11*-*TLR13*, and a pseudogenized *TLR10*). Moreover, the tree shrew *TLR8* and *TLR9* genes had undergone positive selection, possibly due to the adaptation of the pathogen challenge (Yu et al., 2016). The expression of the TLRs varied in response to viral infection. The pseudogenization of *TLR10* in the tree shrew and the positive selection signal in the *TLR8* and *TLR9* genes deserve extensive analyses (Yu et al., 2016). In a similar way to the RLRs, further characterization of the tree shrew's unique pattern of TLRs will help us to understand more about this important innate immune pathway and the antiviral response in the context of creating animal models to investigate infections.

## TREE SHREWS AS MODELS FOR STUDYING FUNDAMENTAL BIOLOGICAL FUNCTIONS AND DISEASE MECHANISMS

For decades, there have been many efforts to promote the tree shrew as an experimental animal and for it to replace primates in the study of fundamental biological functions and human diseases (Amako et al., 2010; Cao et al., 2003; Fang et al., 2016; Fitzpatrick, 1996; Fuchs, 2005; Khani & Rainer, 2012; Lee et al., 2016; MacEvoy et al., 2009; Nair et al., 2014; Peng et al., 1991; Pryce & Fuchs, 2017; Su et al., 1987; Veit et al., 2011, 2014; Xu et al., 2013b; Yan et al., 1996; Zhao et al., 2002; Zheng et al., 2014). Indeed, tree shrews have many characteristics that make it a good laboratory animal, such as small body size, low-cost of maintenance, short reproductive cycle and life span, and most importantly, its close relationship to primates (Fan et al., 2013; Zheng et al., 2014). Also, the ethical concerns of using the tree shrew in biomedical research are less controversial than engendered by the use of primates. Here I would like to highlight several areas where studies using the tree shrew have advanced our knowledge about fundamental biological functions and disease mechanisms, as a way of showing the developing importance of the tree shrew.

### Study of tree shrew visual cortex (striate cortex)

Increasing our fundamental knowledge of brain function, including brain circuits and networks, neural mechanism and information processing underlying cognitive function, is one of the core goals of global brain initiatives (Grillner et al., 2016). The tree shrew has great potential for its use as a suitable animal for studying the function of the visual cortex, as previous studies have revealed a close homology between the tree shrew and the macaque (and human) in the area of visual cortex at both the neuroanatomical and the neurophysiological levels (Fitzpatrick, 1996; Muly & Fitzpatrick, 1992; Van Hooser et al., 2013; Veit et al., 2011, 2014). Thanks to the great efforts by Fitzpatrick and colleagues since 1990s, we now have a better understanding of the morphological basis and functional organization of local circuits and connections in the tree shrew visual cortex, and a better understanding of the response properties of neurons in this region (Fitzpatrick, 1996; Lee et al., 2016; Mooser et al., 2004; Muly & Fitzpatrick, 1992; Van Hooser et al., 2013). In particular, a study of the receptive fields of layer 2/3 neurons in the tree shrew visual cortex revealed the distinct arrangement of ON (light-responsive) and OFF (darkresponsive) pathways, with specific topographic constraints of each representation, to construct orderly columnar representations of stimulus orientation and visual space (Lee et al., 2016). These results derived from using tree shrew visual cortex will undoubtedly assist the fundamental research into brain function and disease.

Other interesting aspects from a comparative point of view are the superior memory-related capabilities of the tree shrew as compared to that of rodents, as has been seen for example in performance of novelty preference tasks (Khani & Rainer, 2012; Nair et al., 2014). The recent studies of the presence and distribution of neuropeptides in tree shrew brain and comparative analyses with other species (Ni et al., 2014, 2015; Petruzziello et al., 2012) has laid the biochemical basis for us to learn more about the specific and common features of tree shrew brain. It is very important to highlight all of these diverse and interesting aspects of the tree shrew, in order to promote the acceptance of this animal more widely in neurobehavioral studies.

### Tree shrew disease models

There are two main kinds of tree shrew disease models at present, the induced model and the spontaneous model. For the induced tree shrew model of disease, surgical and/or chemical approaches are used to induce the onset of disease symptoms in the context of similar phenotypes, similar pathological mechanism, and similar clinical efficacy (McGonigle & Ruggeri, 2014; Yao et al., 2015). Hitherto, the tree shrew has been reported to be used successfully as an animal model for a variety of diseases, such as breast cancer (Ge et al., 2016; Xia et al., 2014), alcohol-induced (Xing et al., 2015) or non-alcoholic fatty liver diseases (Zhang et al., 2015, 2016), hepatitis B virus (HBV) infection (Su et al., 1987; Walter et al., 1996; Yan et al., 1996), hepatitis C virus (HCV) infection (Amako et al., 2010; Xu et al., 2007; Zhao et al., 2002), and herpes simplex virus type 1 (HSV-1) infection (Darai et al., 1978; Li et al., 2016), to name a few, albeit some results need further studies to elucidate the underlying mechanism. In previous review papers, there are ample descriptions and literature surveys of the tree shrew disease models (Cao et al., 2003; Xu et al., 2013b). In a recent book entitled "*Basic Biology and Disease Models of Tree Shrews*", which is the second monograph about the tree shrew in this field, there are also many descriptions of tree shrew models of human diseases, including depression, drug addiction, bacterial infection, breast cancer, glioblastoma, and thrombosis. Some of these models were first described there (see Zheng et al. (2014)). To iterate every detail of each tree shrew model is beyond the scope and space of the current opinion paper, and there is indeed no need to do so. However, I would like to highlight the use of the tree shrew as a model for breast cancer to exemplify the benefits and differences of tree shrew models as compared to current rodent models.

The tree shrew has a similar mammary gland to that of the human based on the morphology and structure (Xia et al., 2014) and this has provided the basis for creating a model of breast cancer. The description of spontaneous breast cancer in the tree shrew dates back to the 1960s when it was reported by Elliot and coworkers (Elliot et al., 1966), that a breast cancer was found in a female tree shrew (*Tupaia glis*) belonging to a different species within the genus *Tupaia*. This animal was in a late stage of pregnancy and had a nodular lesion beneath the skin of the right thoracic breast near the nipple (Elliot et al., 1966). Subsequent studies showed that tree shrews are prone to spontaneous breast tumor, with a frequency of around 1% (Xia et al., 2012, 2014). The spontaneous breast cancer reported by Xia and coworkers (Xia et al., 2012) was an intraductal papilloma, in which epithelium cells grow papillarily with an intact basal membrane. This tumor was positive for progesterone receptor (PR), but negative for human epidermal growth factor receptor 2 (HER2, also named as erb-b2 receptor tyrosine kinase 2 (ERBB2)). It had a high frequency of Ki-67 positive staining and few cleaved caspase-3 positive staining cells, suggesting that the malignant cells are highly proliferative and less apoptotic. The further analysis of 18 spontaneous breast cancers (including the one described by (Xia et al., 2012)) showed that these tumors could be classified as intraductal papilloma (22.2%), papillary carcinoma (55.6%), and invasive ductal carcinoma (22.2%) with or without lung metastasis (Xia et al., 2014). Moreover, tree shrew breast cancerous tissue has frequently been shown to have mutations in the *PTEN/PIK3CA* genes, with a mutation spectrum resembling a subset of human breast cancers with the PTEN/PIK3CA mutations, whereas currently no mouse breast cancer model shows this type of cancer. The *PTEN/PIK3CA* genes mutation status was also correlated with the expression of pAKT in the tree shrew tumorous tissue (Xia et al., 2014).

Besides spontaneously developing breast cancer, the tree shrew could be induced to develop breast cancer by using carcinogen treatment (Xia et al., 2014) and lentivirus expressing the polyomavirus middle T antigen (PyMT) oncogene (Ge et al., 2016). Administration of 7, 12-dimethylbenz(a)anthracene (DMBA) induced a breast tumor in around 12% of tree shrews, whereas co-administration of DMBA and medroxyprogesterone acetate had an even higher success rate for inducing tumor (up to 50%) (Xia et al., 2014). However, the shortcoming of this carcinogeninduced breast tumor was also apparent, with a relatively low frequency and a long latency (around 7 months), but this could be overcome by introducing the lentivirus expressing the PyMT oncogene into the mammary ducts (Ge et al., 2016). The latter lentivirus-mediated approach induced mammary tumors within seven weeks in all tree shrews, with the major tumor type being papillary carcinoma. Further analysis of the PyMT-induced mammary tumors showed elevated levels of phosphorylated AKT, ERK and STAT3 in 41%-68% of tumors. Moreover, growth of the mammary tumors was sensitive to Cisplatin and Epidoxorubicin treatment (Ge et al., 2016). All these efforts showed that tree shrews are capable of developing breast cancer by a variety of approaches, including the spontaneous approach, the surgically and chemically induced approach, and the surgically and lentivirus-mediated oncogene induced approach. The tree shrew breast cancer model best demonstrated a subset of human breast cancer with the PTEN/PIK3CA mutations (Xia et al., 2014). The tree shrew breast cancer model can now be used for the testing of drug efficacy and safety and for elucidating the pathogenesis of mammary tumors.

From the above example of tree shrew breast cancer, it is quite obvious that using the tree shrew could overcome some of the shortcomings of the available rodent models for certain diseases. Tree shrew models of human diseases have been shown to have many benefits, albeit some of the models need further improvement for repeatability, stability, and uniformity. Moreover, analysis of tree shrew disease models will also provide a novel molecular basis to study individual human diseases, such as the susceptibility to stress (Fang et al., 2016). However, it should be mentioned that so far, the present tree shrew models have not demonstrated a unique advantage in drug development or succeeded in leading to any significant scientific discovery. The only exception to this may possibly come from the identification of sodium taurocholate cotransporting polypeptide as a functional receptor for HBV and hepatitis D virus (HDV) infection (Yan et al., 2012), which was triggered by the observation that the tree shrew and its hepatocytes could be infected by HBV (Su et al., 1987; Walter et al., 1996; Yan et al., 1996). I expect that a genome-based approach may be more helpful for the design of tree shrew models of human diseases. Namely, based on the characterization of the tree shrew's unique genetic features and common genes and pathways, instead of "trial and error", will be a more efficient method to create a new model of tree shrew and to predict the validity of the model. A convenient way to start is by looking at the genes and pathways of the tree shrew by searching the tree shrew genome database (www.treeshrewdb.org) (Fan et al., 2014a) to find items which have a close similarity to their human counterparts. But to overcome the complications of the heterogeneous genetic background of various captured wild tree shrews and to achieve a uniform response of each animal during the creation of animal models and drug tests, there is a pressing need to establish tree shrew inbred lines.

### Genome editing of tree shrew

**Figure 2:**
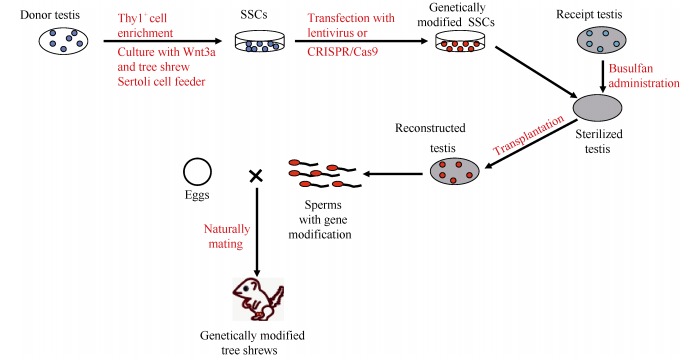
Schematic procedure for making a transgenic tree shrew by using the spermatogonial stem cells based on the study by Li et al. (2017)

The recent advances in genetic manipulation techniques, in particular CRISPR/Cas9 technology (Cong et al., 2013; Hsu et al., 2014; Luo et al., 2016; Mei et al., 2016; Shao et al., 2016), have provided methods whereby it is possible to perform genome editing of any non-model animal and to make genetically modified animal models. Due to the lack of knowledge about the reproductive biology and assisted reproduction technologies in the tree shrew (Yan et al., 2016), gene editing methods using one-cell embryos or embryonic stem cells, which are commonly used in rodents (Wang et al., 2015) and primates (Guo & Li, 2015; Niu et al., 2014), have hitherto been unavailable for the tree shrew. This limitation has also hindered the wide acceptance of the tree shrew as an important laboratory animal. This disadvantage was luckily resolved by our recent success based on the isolation of tree shrew spermatogonial stem cells (SSCs) and the establishment of a culture system for these SSCs (Li et al., 2017). In this pioneer study, we solved the two key technical obstacles for establishing a line of SSC: identification of the necessary supplements for the culture medium and the isolation of suitable feeder cells for producing SSCs. In brief, we used the thymus cell antigen 1 (Thy1^+^) to enrich tree shrew SSCs, followed by transcriptomic analysis to massively identify the activated signaling pathways during the differentiation of cultured SSCs. We then attempted to optimize the culture medium by supplying additional protein that plays a role in the activated signaling pathway, such as Wnt3a protein in the Wnt/β-catenin signaling pathway. This strategy was very successful in maintaining the survival of tree shrew primary SSCs. The other technical trick involves the use of tree shrew Sertoli cells, but not mouse embryonic fibroblasts, as feeder for the expansion and longterm culture of tree shrew SSCs. The expanded SSCs could then be transfected with the lentiviral vector containing the enhanced green fluorescent protein (EGFP), and the positive SSC clone was expanded and transplanted into the testes of sterilized adult male recipients, which were created by treating adult male tree shrews with Busulfan to eliminate the preexisting germ cells. The expanded SSCs were capable of continuous spermatogenesis after transfection with EGFPexpressing virus and transplantation into recipient males, and EGFP-tagged sperms generated viable transgenic tree shrews (Figure 2) (Li et al., 2017). By way of example, we showed that these SSCs were suitable for the CRISPR/Cas9-mediated knockout of the *APP* (amyloid beta precursor protein) gene, with a reasonably high efficiency in single SSC cells (17/70=24.3%) (Li et al., 2017), although no living offspring have as yet been created at the time of publishing our paper. This is the first study of a successful genetic manipulation of tree shrew. We believe that more genetically modified tree shrews will be produced in the near future by using this SSC-based gene editing strategy. Currently, we are working on the creation of transgenic tree shrews with human APP and PSEN1 mutations that are causal for AD. We expect that the tree shrew AD model will be superior to the currently available rodent AD models.

## FUTURE PERSPECTIVE FOR TREE SHREW USAGE IN BIOMEDICAL RESEARCH

Based on the above descriptions, it is no doubt of the tree shrew's superiority over rodents for studying certain human diseases and understanding the neural mechanism of brain function resulting from specific aspects of genetic makeup or lifestyle. In particular, the tree shrew has the top three benefits of our human/monkey research partnerships in biomedical research: safety, efficacy, and greater predictability. The more we know about tree shrew, the more success we can expect to have. The recent release of the tree shrew genome database (www.treeshrewdb.org) has provided for easy access to the tree shrew genome data (Fan et al., 2014a). We are currently re-sequencing more tree shrew individuals, in particular from inbred lines, with the intention of refining the quality of the tree shrew genome and improving our understanding of the genomic diversity of this animal. The re-sequencing data will be deposited into the tree shrew genome database. We have also collected and compiled the available transcriptomic data from the tree shrew, and will frequently update the information at the webserver. Following the genome-based approach to retrieve more information regarding the genetic makeup of tree shrew, especially for the nervous and immune systems, it would be easier and safer to make a valid design of a tree shrew model of human diseases and to uncover the underlying mechanisms.

With the successful gene manipulation of the tree shrew (Li et al., 2017) and by the use of cutting-edge techniques, e.g., two-photon imaging of GCaMP6 calcium signals for neurons in visual cortex (Lee et al., 2016), there is only one big obstacle remaining for a wider usage of tree shrew, that is, the establishment of inbred lines. At present we are attempting to create the tree shrew inbred lines at the Kunming Institute of Zoology (KIZ), Chinese Academy of Science (CAS), and we have successfully achieved F4 offspring by sibling mating although the population size is still very small. We have every reason to believe that the tree shrew, with a tag of "made in China" or "created in China", will become reality in the coming years.

## ACKNOWLEDGEMENTS

I thank Ian Logan (22 Parkside Drive, Exmouth, Devon, UK), Wai-Yee Chan (The Chinese University of Hong Kong) and three anonymous reviewers for helpful comments on the early version of the manuscript. I thank Ping Zheng (KIZ, CAS) for helping with the preparation of Figure 2, Ling Xu and Dan-Dan Yu (KIZ, CAS) for making Figure 1. The opinions expressed in this paper represent my personal views. I would like to apologize to those colleagues whose work was not mentioned or elaborately represented in this opinion paper as I was not making a comprehensive literature survey for all tree shrew studies and/or because of my ignorance and negligence.
